# How doctors make themselves understood in primary care consultations: A mixed methods analysis of video data applying health literacy universal precautions

**DOI:** 10.1371/journal.pone.0257312

**Published:** 2021-09-21

**Authors:** Jennifer V. Byrne, Katriina L. Whitaker, Georgia B. Black

**Affiliations:** 1 Applied Health Research Department, University College London, London, United Kingdom; 2 School of Health Sciences, University of Surrey, Guildford, United Kingdom; Zurich University of applied sciences, School of Health Professions, Leitung Forschunsstelle Gesundheitswissenschaften/Head of health sciences research, SWITZERLAND

## Abstract

**Purpose:**

To mitigate the health risks that result from low health literacy and difficulty identifying patients with insufficient health literacy, health organizations recommend physicians apply health literacy universal precaution communication skills when communicating with all patients. Our aim was to assess how health literacy universal precautions are delivered in routine GP consultations, and explore whether there were differences in how GPs used universal precaution approaches according to areas of deprivation in England.

**Methods:**

This was a mixed methods study using video and interview data. Ten physicians conducted 217 consultations in primary care settings with adults over 50 years old between July 2017 and March 2018 in England. Eighty consultations (N = 80) met the inclusion criteria of new or persisting problems. Descriptive quantitative analysis of video-recorded consultations using an observation tool and qualitative thematic analysis of transcribed scripts. Meta-themes explored differences in physicians’ communication by areas of deprivation.

**Results:**

Descriptive statistics showed physicians used a caring tone of voice and attitude (n = 73, 91.3%) and displayed comfortable body language (n = 69, 86.3%) but infrequently demonstrated profession-specific health literacy universal precaution communication skills, such as the teach-back technique (n = 3, 3.8%). Inferences about physicians’ communication from qualitative analysis converged with the quantitative findings. Differences in physicians’ communication varied according to areas of deprivation.

**Conclusions:**

Physicians need health literacy universal precautions communication skills to improve population health.

## Introduction

Making sure patients understand what to do in response to health information is universally important in the provision of healthcare services. Healthcare practitioners need to take responsibility as communicators of health information to actively build and empower patients’ health literacy [1,2], defined by international health bodies as an individual’s ability to obtain, understand, and use information and services to manage their health [3–6].

Mitigating health illiteracy has the potential to create a step-change in health outcomes with its high prevalence in North America, Australia, and Europe (29%-62%), particularly in the context of global financial austerity, an ageing population and increasing non-communicable diseases[3,7–13]. Lower health literacy is associated with lower socioeconomic status, older age and financial deprivation [9,13]. Nearly every major health outcome indicator is associated with low health literacy, such as poor medication adherence, poor chronic disease self-management, worse disease outcomes, and increased healthcare costs [1,11,12,14–17]. Low health literacy is a preventable and modifiable risk factor of socioeconomic disparities in health [18]; yet, health inequities persist [5,14].

While validated tools exist to measure a patient’s health literacy [13,19,20], significant problems prohibit their practical application: individual health literacy changes with health topics, over time, and may be influenced by emotions and cognitive impairment affecting their ability to comprehend health information [7,16,20]. In addition, research demonstrates that physicians cannot identify patients with low health literacy, routinely overestimate a patient’s health literacy, and overestimate their own communication abilities [4,21–25].

One approach to mitigating low health literacy is to use health literacy universal precautions: this stipulates that all patients should be treated as at-risk of misinterpreting health information by simplifying communication and checking comprehension [26]. Leading health organizations and peer-reviewed journals endorse the evidence-based tools and resources included in the USA Agency for Healthcare Research and Quality’s (AHRQ) Health Literacy Universal Precautions Toolkit [AHRQ Toolkit; 4,5,22]. The AHRQ Toolkit recommends practitioners avoid medical jargon by communicating clearly using plain language [20,22,26,27]. It endorses the teach-back technique, where the practitioner assesses the patient’s understanding by asking them to explain what they were told in their own words [12,22]. Finally, the toolkit suggests that practitioners create a shame-free environment to build the patient’s confidence to care for themselves [22].

Despite universal understanding of the importance of health literacy, communication models guiding physicians [23,28,29], and evidence-based approaches to apply health literacy universal precautions, little is known about how physicians’ language and behaviour fulfils those precautions. Our aim was to assess how aspects of GP communication such as speech, vocabulary, body language, printed materials fulfil health literacy universal precautions in routine GP consultations. Given the association between lower health literacy and lower socioeconomic status, we also explored whether there were differences in how GPs used universal precaution approaches according to areas of deprivation in England.

## Materials and methods

### Video data collection

This was a mixed methods study using qualitative data including video-recordings and verbatim transcripts from a previous study [30] collected from July 2017 to March 2018 at seven primary care practices in England. A convenience sample of physicians provided informed, written consent. Patients seen by the participating physicians who were over the age of 50, spoke English, and capable of consenting were eligible to participate in the study. A member of the research team, with no relationship to the participants, approached eligible patients in the waiting room with 74% providing informed, written consent. Physicians and patients completed demographic questionnaires and were video-recorded during consultations. Only study participants were present and the physicians controlled the filming. London Chelsea Research Ethics Committee (REC Ref: 17/LO/0270) granted ethics approval for the original study and subsequent analyses [30].

A mixed methods study design was used to answer the research question and objectives using data related to new and persistent problems only [30,31]. The qualitative and quantitative components were analysed separately and triangulated to substantiate themes. The research met the Consolidated Criteria for Reporting Qualitative Studies (COREQ) [32]. This study uses highly identifiable video data, making it unsuitable for open sharing. Anonymised transcripts of the video may be shared by individual application to the authors. Video data may also be available for secondary analysis, dependent on appropriate ethical approvals and author consideration.

### Qualitative thematic analysis

From June through July 2019, transcribed scripts of the video-recorded consultations were analysed in Nvivo Software following the principles of thematic analysis [33]. One researcher (JB) noted their impressions on the transcript of each consultation as part of the process of familiarisation with the data, focussing on aspects of the GP’s and patient’s language which could be relevant to health literacy. After 7 transcripts had been annotated in this way, the research team met to discuss their impression and formulate some structured codes to proceed with the rest of the dataset. While the coding was open and inductive, the items of the AHRQ Toolkit were prominent in our interpretation of the data in order to serve our research aims. JB’s background included both professional and academic understanding of health literacy concepts. We would therefore characterise this as an inductive-deductive hybrid approach to thematic analysis [34]. For example, indications of whether or not the patient understood the GP, and the type of language used by the GP. JB met regularly with the co-authors to discuss initial impressions and to gain robust feedback about her assumptions and interpretations [35]. Another researcher KW reviewed the coded transcripts and code lists for face-validity to ensure the codes related to the research question and objectives. No changes to the codes or approach were recommended at that time. JB inductively coded the remaining transcribed scripts in alphabetical order. The authors collectively developed a coding manual which included the code name and a definition of what it concerns. drawing on methods for analysing verbatim conversation such as identifying skilled conversation [33,36]. The coded data were inputted into Microsoft Excel to facilitate comparisons between different excerpts from consultations and interviews. All authors used the Excel tables to review the development of themes (i.e. patterns and storylines within the data) in relation to the coded data and then defined and named the themes. All authors contributed to writing the themes for publication incorporating quotations from the videos and interviews.

### Application of the AHRQ Toolkit as a coding tool

From June to July 2019, the video sequences of consultations were evaluated using the *Always Use Teach-back*! *Teach-back Observation Tool (Observation Tool)* to evaluate physicians’ attempts to be understood, chosen due to its inclusion in the AHRQ Toolkit [4,5]. This created numerical scores that allowed the data to be analysed quantitatively. The Observation Tool was designed to help observers evaluate physicians’ communication to aid in the establishment of consistent health literacy universal precaution communication habits [22,37]. The Observation Tool was absent when the AHRQ Toolkit underwent validity testing; to our knowledge, no other validated health literacy assessment tools exist that evaluate GPs’ health literacy communication via observation. [17,19,20,22,38–40].

Each consultation was evaluated against the Observation Tool criteria using nominal, categorical variables (Table 1). Some of the Observation Tool criteria were clear whereas other criteria required inductive interpretation to evaluate the consultations. For example, parameters for the use of plain language were defined based on best-practices from the AHRQ Toolkit [22,37]. The Observation Tool items were refined through discussion with a practising physician and another author (KW) to improve the face validity of the tool and refine the variable. For example, we decided whether some items should be in binary or Likert scale format.

**Table 1:** Adapted observation tool [37].

Criteria		Yes	No	Not Applicable
Did the physician:	Use a caring tone of voice and attitude?			
	Display comfortable body language?			
	Use plain language?			
	Ask the patient to explain in their own words what they were told to do?			
	Use non-shaming, open-ended questions?			
	Avoid asking questions that patients can answer with a “yes” or “no”?			
	Take responsibility for making sure they were clear?			
	Explain and check again if the patient is unable to teach-back?			
	Use reader friendly print materials?			
	Document patient’s response to teach-back?			

The consultations were evaluated by JB in alphabetical order and according to the parameters. A small sub-sample (n = 9) was scored by another author (KW) at the beginning of the analysis process to test the usability of the scoring system, although a formal interrater reliability score was not generated. No changes were made following this process. Other researchers were not permitted due to the strict data security requirements relating to the video-recordings. Robust discussions and peer debriefing was used through the analysis phase to resolve queries about scoring, interpretation of ambiguous or unclear aspects of the data, and increase the reliability of the analysis. Scores were entered manually into an Excel spreadsheet and analyzed by the authors after. Descriptive statistics were used to examine the proportion of consultations where physicians displayed Observation Tool criteria and to examine the proportional differences in physicians’ communication in low and high deprivation areas according to the United Kingdom’s (UK) Index of Multiple Deprivation (IMD) [41]. IMD provides a set of relative measures of deprivation across England, based on seven different domains; income, employment, education, health and disability, crime, barriers to housing and services and living environment. We defined high deprivation as GP practices falling within the lowest five deciles of deprivation (1–5) and low deprivation as those falling within the top two deciles (9 and 10) [Ministry of Housing, Communities & Local Government 42].

### Triangulation and meta-themes

To enhance the validity of the research, both qualitative and quantitative findings were studied after initial analysis through an adapted triangulation approach [43,44]. Through the triangulation process, meta-themes were identified that encompassed the findings from both research methods. The authors extensively discussed the meta-themes ensuring higher level interpretations were valid, consistent, and insightful.

## Results

### Descriptive characteristics

Ten clinicians took part in the study. Of 217 consented patients, 200 completed videos without technical issues, and of these, a final subsample of 80 consultations were identified where patients presented new or persistent problems (N = 80). New or persistent problems were defined as those the patient discussed with their doctor for the first time and where no diagnosis had been formulated yet (e.g. lump, hair loss). A full list of presenting problems has been published previously [30]. The data set (N = 80) was comprised of 57 (71.0%) consultations that took place in three clinics located in low deprivation areas (deciles 9 & 10). Twenty-three (29.0%) consultations took place in four clinics located in high deprivation areas (deciles 1–5). Table 2 and Amelung et al. [30] provide descriptive characteristics of the data set.

**Table 2:** Adapted descriptive characteristics of physicians and patients included in the final data set [32].

Characteristic	Doctors (N = 10)	Patients (N = 80)
Sex n (%)		
Female	3 (30.0)	46 (57.5)
Age (years) mean (SD, min-max)	48.2 (10.2, 32–60)	66.5 (11.3, 50–96)
Years since accreditation as a doctor (SD, min-max)	15.9 (11.5, 2–32)	Not applicable

### Quantitative findings

**Fig 1:**
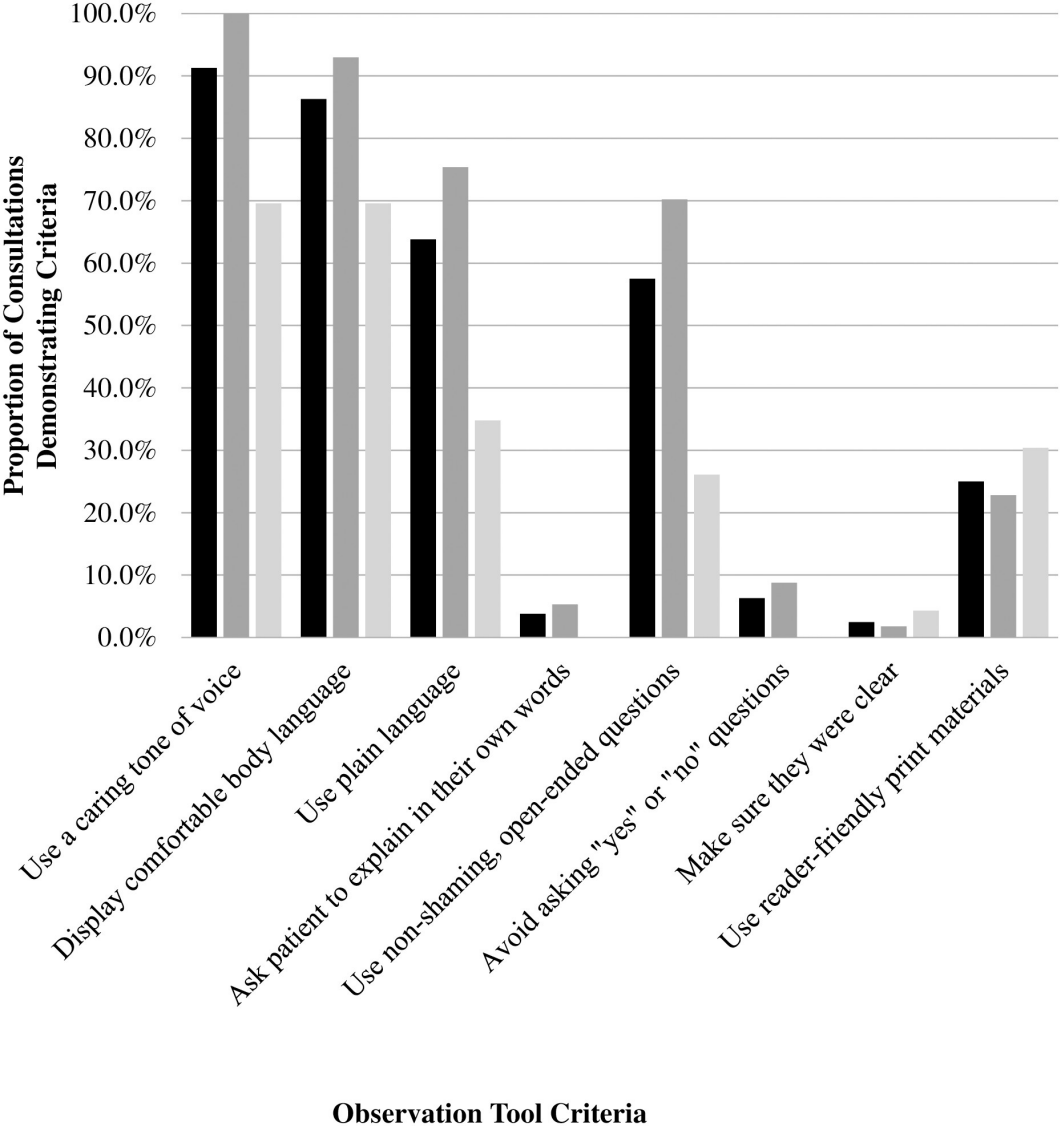
Proportion of consultations where physicians used health literacy universal precautions to communicate with patients in aggregate format and stratified by areas of deprivation. * Black bars represent the proportion of aggregate consultations demonstrating criteria (N = 80). ^†^ Dark gray bars represent the proporition of consultations demonstrating criteria in low deprivation areas (n = 57). ^‡^ Light gray bars represent the proporition of consultations demonstrating criteria in high deprivation areas (n = 23). ^§^ Criteria adapted from Observation Tool (Asan & Montague, 2014). ^‖^ The following Observation Tool criteria were excluded from the graph because they did not apply to the majority of consultations: 1) explain and check again if the patient is unable to teach-back; 2) document the patient’s teach-back response; and 3) include present family members or caregivers.

The aggregate results and results stratified according to deprivation areas are presented in Fig 1. The consultations rarely exemplified physicians asking the patients to explain what they were told to do in their own words (n = 3, 3.8%) or the physicians checking to make sure they were clear (n = 2, 2.5%). When physicians did not check with patients to make sure they were clear during the consultation, certain Observation Tool criteria could not be accomplished and were coded as not applicable according to the coding parameters. Nearly all of the consultations were coded as not applicable for the following criteria: explain and check again if the patient could not teach-back (n = 78, 97.5%) and document the patient’s teach-back response (n = 80, 100.0%).

Differences in physician communication were examined according to areas of deprivation. Regardless of deprivation areas, physicians routinely demonstrated effective interpersonal communication. In areas of low deprivation, physicians nearly always used a caring tone of voice (n = 57, 100.0%) and displayed comfortable body language (n = 53, 93.0%). In areas of high deprivation, physicians frequently used a caring tone of voice while displaying comfortable body language (each n = 16, 69.6%).

Specific health literacy universal precaution communication skills varied widely among physicians according to areas of deprivation. In areas of low deprivation, physicians demonstrated more plain language (n = 43, 75.4%) compared to physicians conducting consultations in areas of high deprivation (n = 8, 34.8%). There was some evidence of physicians in areas of low deprivation that sporadically asked patients to explain what they were told to do in their own words (n = 3, 5.3%) and intermittently avoided asking questions that patients could answer with a “yes” or “no” (n = 5, 8.8%). Whereas, physicians in high deprivation areas made no attempts to ask patients to explain what they were told to do in their own words and did not avoid asking questions that patients could be answered with a “yes” or “no” (each, n = 0, 0%).

### Qualitative analysis leads to converging meta-themes

The findings from the Observation Tool were triangulated with the qualitative analysis resulting in the emergence of three converging meta-themes exemplified with case studies with differences by deprivation area considered throughout. The meta-themes exemplify how doctors make themselves understood and the struggles they have in helping patients to understand health information. This contradicts universal health literacy precautions and limits patients’ ability to understand.

#### How doctors make themselves understood

The findings demonstrated physicians’ attempted to use select health literacy universal precautions to help patients understand health information. For example, physicians used plain language (e.g. flu) and tools (e.g. images), asked patients open-ended questions, and conveyed interest through non-verbal communication. These observations were not isolated to select physicians or areas of deprivation.

Table 3 exemplifies the types of conversations physicians had with patients using plain language. In the low deprivation area case study, the physician used plain language to investigate the patient’s health issue and explained their tongue’s anatomy. In the high deprivation area case study, the patient asked the physician to explain arthritis. The physician used plain language to describe the symptoms of arthritis. According to the AHRQ Toolkit, both case studies would reflect optimal health literacy universal precaution communication skills if the physician checked that the patient understood the physician’s use of plain language by asking a question that prompted the patient to teach-back the information.

**Table 3:** Examples of conversations where the physicians used plain language.

Low Deprivation Area	High Deprivation Area
*The tongue? It looks okay, let me see again*. *Okay, it’s just the normal taste buds on your tongue, so let me show you. On your tongue there are different lumps, this is normal*. Normal, yeah? *The front of the tongue is little lumps and at the back of the tongue there are big lumps, they’re the taste buds*. *So, it’s normal*.	What is-that’s not arthritis, is that right? *Well, it could be*. Is arthritis soreness, is that what you get? *Yes, it’s sore, it can be stiff, it can be swollen*.

* Regular font represents patient speaking.

^†^*Italic font* represents physician speaking.

^‡^Underlined font represents use of plain language.

These case studies demonstrate a finding echoed across the analysis (Fig 1), which indicates that physicians in areas of low deprivation tend to use plain language without requiring a prompt from the patient, which differs from physicians in areas of high deprivation. These cases demonstrate physicians’ attempt to apply health literacy universal precaution communication skills to aid the patient’s understanding and use of health information but contextual factors about the patient population affect physicians’ attempts.

#### Doctors struggle to help patients understand health information

This theme demonstrates that physicians experienced particular challenges in mitigating inadequate health literacy when benchmarked against health literacy universal precautions. This was exposed in the data when patients indicated that they did not understand health information. For example, physicians resort to asking “yes” or “no” questions, do not check whether they were clear, and do not use teach-back techniques to check the patient’s understanding. Table 4 exemplifies the types of conversations physicians had with patients when patients indicated they do not understand health information.

**Table 4:** Examples of conversations where the patients misunderstand health information.

Low Deprivation Area	High Deprivation Area
*And what was the ones that you didn’t like?* Oh! *Oh, it’ll be in our system, let me look it up*. I don’t know. Dr. [xxx] and Dr. [xxx] gave it… *Was it Trimethoprim or Nitrofurantoin?* I don’t know what they’re called. *Oh yeah Nitrofurantoin okay*. *So yes, the microbiologists, the lab that analyse all these samples, always are recommending to us Nitrofurantoin first, but what did it give to you that’s made you…?* Well, straight away I had no problem, but the next day, or the next day after, I was so, I couldn’t, I couldn’t erm…I can’t remember, I can’t say what I want to say. *Okay, don’t worry*. But those work, sometimes it’s slowly, but these… *Yeah, okay, well I’ll give you those ones, then, okay*.	*Could I give you a box to take on top of the ones that you are already taking- the package, you think you’d remember to do that?* They give me…they give me every two weeks. *Yeah*. Two boxes. *Oh I see, okay*… You see. *So if I gave you another tablet-eh a-a separate box* Ah! *To take an extra tablet in the morning* Okay. *Do you think you’d remember that?* I take them for a month *Well, so you have the tablets that you are normally taking in the morning- you take it-you take an extra one*. Ye-yes, I take extra one. Is-is eh is for the-(touches her leg) *For the swelling, yeah*. That one I take it…one every week. *Oh, I see, that’s for the bones, that one. So this would be an extra one you take every day in the morning* No, I don’t take- *Yes, but I-I would provide you with a new medicine*. You know…you know what colour is it? *I’ve ehm…I’ve- no, I don’t know but I could ask the pharmacist to find out…* Eh, yes. *Yeah?…If I send the box to the chemist with the cream for your legs and the gel for your hand, you would just take one of those new tablets in the morning with all your other tablets…okay?* Okay. *Yeah?* Take one in the morning. *That’s right*. With the others. *Yes. And it’ll be on the box*. Aah. *What I’ll do is I’ll give you a ring next week to make sure you’re getting on okay with the tablets, okay?*

* Regular font represents patient speaking.

^†^*Italic font* represents physician speaking.

^‡^Underlined font represents the physicians’ attempts to make themselves understood.

In the low deprivation area case study (Table 4), the physician tried to understand what medications worked for the patient in order to refill prescriptions. The physician asked a combination of non-shaming, open-ended questions and questions that the patient could answer with a “yes” or “no” in an attempt to identify the medication that worked best for the patient. The physician used the medical records’ prescription history to ask the patient about certain medications but the patient indicated they did not remember. The physician did not continue to engage the patient but instead decided for them what prescription to renew.

In the area of high deprivation case study (Table 4), the physician tried to understand whether or not the patient would take a new medication. The patient repeatedly indicated that they did not understand. The physician repetitiously explained the new medication regiment and continually asked questions that could be answered with a “yes” or “no” to check the patient’s comprehension. Ultimately, the physician was not confident the patient understood or could act on the new medication regiment.

There was minimal use of profession-specific health literacy universal precaution communication skills with minimal variation according to areas of deprivation, which is exemplified in Fig 1. For example, physicians rarely asked patients to explain what they were told to do in their own words in low deprivation areas (n = 3, 5.3%) and never demonstrated this in high deprivation areas (n = 0, 0%). These case studies demonstrate a finding echoed across the analysis indicating that physicians do not consistently display the necessary, profession-specific communication skills that enable them to communicate effectively when patients indicate they do not understand health information.

#### Contradicting health literacy universal precautions impacts the patients’ ability to understand health information

This theme demonstrates physicians’ behaviours that contradict health literacy universal precautions. Examples include language that could make the patient feel ashamed, talking over the patient to limit the conversation, and ignoring the patient’s input when not explicitly solicited.

In a low deprivation area case study, the patient expressed concerns about getting a chest infection. The physician responded by stating, *“I-I just wonder whether this-this-this worry that you’ve got about…picking up pneumonia and so on*, *ehm*, *whether some of that worry energy should be diverted into giving up smoking because twenty or 35 years of smoking 20 a day is a lot*, *and you know-it has consequences*.*”* Dismissing the patient’s concern may have provoked feelings of shame, and prompted the patient to defend and explain their difficulty quitting. Ultimately, the physician continued in this vein by stating, *“Well if it was easy to give up*, *I suppose nobody would be hooked on it*, *would they*?*”* This demonstrates that physician are juggling multiple agendas, both attempting to address health needs such as smoking cessation, but in a manner that could indirectly encourage the patient not to act on the doctor’s concerns.

In a high deprivation area case study, the patient requested an automatic medication refill be stopped but could not remember what prescription it was. The patient started to ask the physician to discuss the medications again but the physician interrupted the patient stating, *“I just told you*, *I just went through all the ones that are on there*.*”* The patient agreed with the physician after it was indicated that they should have remembered their prior conversation about current medications.

These findings were represented across the analysis illustrating that physicians in both low and high areas of deprivation could potentially put the physician-patient relationship at-risk by lacking health literacy universal precaution communication skills.

## Discussion

This is the first study to map physician behaviours during consultations against the Observation Tool endorsed in the AHRQ Toolkit [22]. This research revealed that while physicians consistently exercised some precautions during consultations, such as displaying comfortable body language, these were based on basic communication skills intrinsic to an individual’s personality [23,45]. Specific communication skills unique to physician-patient conversations, such as the teach-back technique, were not routinely observed likely because they are not acquired through basic or interpersonal communications. The importance of the teach-back technique has been highlighted as a critical component to reducing physician-patient miscommunications [46], which is currently lacking in different arenas of clinical practice [47]. This research complements previous insights that recommend practitioners be better equipped with communication skills to communicate effectively with a low health literacy audience since physicians will not acquire these skills during routine practice [16,25,48]. Across deprivation areas, physicians often contradicted health literacy universal precaution communication skills when they struggled to help the patient understand health information. When physicians are not taught specific, and perhaps less intuitive, communication skills to help make themselves understood, they may not demonstrate health literacy universal precaution communication skills spontaneously.

Communication skills specific to physician-patient conversations require training and practice to be seamlessly integrated into habitual communication approaches applied by physicians. For over 15 years, the UK General Medical Council has required physicians to be able to demonstrate clear, effective communication with patients [49,50]. Moreover, the UK Council of Clinical Communication Skills Teaching in Undergraduate Medical Education explicitly states doctors need the skills to check patient’s understanding [51]. Therefore, one may expect the routine observation of physicians demonstrating effective communication skills to help make themselves understood. Lack of routine observation of effective communication skills is exacerbated by the evidence exemplifying physicians will not acquire these skills during clinical practice [48]. These findings emphasize that in order for each consultation to successfully enable patients’ understanding, physicians need to be specifically trained on health literacy universal precautions.

Use of health literacy universal precautions varied according to areas of deprivation. For example, nearly twice as much use of plain language was observed in low deprivation areas compared to high deprivation areas. This is counterintuitive and not easily explained, although in line with the Inverse Care Law: that the quality of care is inversely related to the need of the population served [52]. This risks exacerbation of adverse health outcomes for an already vulnerable population [9,13,53]. This may be related to added challenges in delivering universal health precautions in more deprived areas such as higher co-morbidities, complex consultation needs and reduced practice resources. Enhanced training and equipment with profession-specific communication skills should therefore be given precedence in these areas.

A key strength in this study is the triangulation of qualitative and quantitative findings. Viewing the video-recorded consultations and reading the transcribed scripts allowed this investigation to interpret both verbal and non-verbal communication in clinical settings, which is enlightening given physicians overestimate their communication abilities [23].

While other studies demonstrated the effectiveness of the AHRQ Toolkit, this is the first study to evaluate physicians’ communication using the Observation Tool in clinical practice [20,22,26,39,54]. The quantitative findings from the Observation Tool converged with the qualitative findings which increases our confidence in the effectiveness of the Toolkit as an Observation Tool. However, we observed that items such as the use of shaming language are hard to interpret. For example, the GP may use language that provokes a defensive response from the patient, despite aiming to persuade the patient do something for their own benefit [55]. The Observation Tool required inductive interpretation of evaluation criteria, which could be clarified to increase consistency among observers. Although descriptive rather than inferential statistics were conducted to meet the study aims, future work with a validated tool and larger samples could involve more in-depth analyses. The Observation Tool was created in the USA and critically evaluated for the first time in the UK; therefore, there may be international differences in its applicability warranting further investigation.

One limitation of our dataset is the gender imbalance between the clinicians (30% female) and patients (57% female). Demographic concordance between GP and patient is a known facilitator for communication [56]. A key limitation of this research stems from lack of protocols that enable standardized approaches to mixed methods research and exercising reflexivity while conducting qualitative research [35,57]. The authors attempted to mitigate this by adhering to the COREQ [32]. Despite exercising iterative reflexivity, JB’s familiarity with health literacy concepts influenced coding. However, the AHRQ Toolkit emphasizes the Observation Tool should be used as a mechanism to guide coaching of consistent communication habits implying the observer should possess familiarity with health literacy [22].

The previous study responsible for recruiting participants and collecting the data was subject to selection bias. Physicians that agreed to participate have different characteristics than those that opted out [58]. Physicians that did not agree to participate are more likely to be older, less well-qualified, and less likely to be viewed by patients as helpful [59]. Therefore, the findings could overestimate the proportion of physicians applying health literacy universal precautions when conversing with patients. Although small, the sample size of physicians was deemed sufficient due to the large/diverse number of consultations analysed. Finally, the eligibility criteria from the original study restricted the sample to people 50 years or older. Low health literacy affects people of all ages but evidence demonstrates older populations are more likely to experience severe consequences from low health literacy [15].

These findings provide actionable insights. Health literacy universal precautions needs to be seamlessly integrated into communication models. Physicians need to be better equipped with health literacy universal precaution communication skills to adapt to the needs of a low health literacy audience. Training physicians in high deprivation areas to reduce inequities needs to be prioritized. Physicians that communicate less effectively exacerbates their low health literate patients’ vulnerability to poor health outcomes. With the benefits of using video-recorded observational data in clinical settings becoming more widely acknowledged and accepted, further research should focus on validating an Observation Tool that can be used to evaluate physicians’ communication as it relates to health literacy universal precautions [60]. These findings underscore the importance of translating endorsed health literacy universal precaution approaches into routine clinical practice to improve population health.

## Supporting information

S1 File (DOCX)
