# Supplementary material for: How doctors make themselves understood in primary care consultations: A mixed methods analysis of video data applying health literacy universal precautions
Source: PLoS One. 2021 Sep 21;16(9):e0257312. doi: 10.1371/journal.pone.0257312 (PMC8454934; doi:10.1371/journal.pone.0257312)
Supplement: S1 File — (DOCX) [file pone.0257312.s001.docx]

## Appendix A: Consolidated Criteria for Reporting Qualitative Studies 32-item checklist and study adherence

| **#** | **Item** | **Guide Questions/ Description** | **This Study’s Adherence** |
| --- | --- | --- | --- |
| Domain 1: Research team and reflexivity | | | |
| 1 | Interviewer/facilitator | Which author/s conducted the interview or focus groups? | Not applicable. |
| 2 | Credentials | What were the researcher’s credentials? | Title page, page 1 |
| 3 | Occupation | What was their occupation at the time of the study? | Title page, page 1 |
| 4 | Gender | Was the researcher male or female? | Title page, page 1 |
| 5 | Experience and training | What experience or training did the researcher have? | Methods section, pages 8 |
| 6 | Relationship established | Was a relationship established prior to study commencement? | Methods section, page 8 |
| 7 | Participant knowledge of the interviewer | What did the participants know about the researcher? | Methods section, page 8 |
| 8 | Interviewer characteristics | What characteristics were reported about the interviewer/facilitator? | Not applicable. |
| Domain 2: Study Design | | | |
| 9 | Methodological orientation and  Theory | What methodological orientation was stated to underpin the study? e.g. grounded theory,  discourse analysis, ethnography, phenomenology, content analysis | Methods section, page 7-8 |
| 10 | Sampling | How were participants selected? | Methods section, page 7 |
| 11 | Method of approach | How were participants approached? | Methods section, page 7 |
| 12 | Sample size | How many participants were in the study? | Methods section, pages 7 |
| 13 | Non-participation | How many people refused to participate or dropped out? Reasons? | Methods section, page 7 |
| 14 | Setting of data collection | Where was the data collected? | Methods section, page 7 |
| 15 | Presence of non-participants | Was anyone else present besides the participants and researchers? | Methods section, page 7 |
| 16 | Description of sample | What are the important characteristics of the sample? e.g. demographic data, date | Methods section, page 7 |
| 17 | Interview guide | Were questions, prompts, guides provided by the authors? Was it pilot tested? | Not applicable. |
| 18 | Repeat Interviews | Were repeat interviews carried out? If yes, how many? | Not applicable. |
| 19 | Audio/visual recording | Did the research use audio or visual recording to collect the data? | Methods section, pages 7 |
| 20 | Field notes | Were field notes made during and/or after the interview or focus group? | Not applicable. |
| 21 | Duration | What was the duration of the interviews or focus group? | Not applicable. |
| 22 | Data Saturation | Was data saturation discussed? | Not applicable. |
| 23 | Transcripts returned | Were transcripts returned to participants for comment and/or correction? | Not applicable. |
| Domain 3: Analysis and Findings | | | |
| 24 | Number of data coders | How many data coders coded the data? | Methods section, page 8 |
| 25 | Description of the coding tree | Did authors provide a description of the coding tree? | Methods section, pages 8 |
| 26 | Derivation of themes | Were themes identified in advance or derived from the data? | Methods section, pages 8 |
| 27 | Software | What software, if applicable, was used to manage the data? | Methods section, pages 7-8 |
| 28 | Participant checking | Did participants provide feedback on the findings? | Not applicable. |
| 29 | Quotations presented | Were participant quotations presented to illustrate the themes / findings? Was each quotation identified? e.g. participant number | Throughout Results section, e.g. page 14 |
| 30 | Data and findings consistent | Was there consistency between the data presented and the findings? | Results section, pages 11-15 |
| 31 | Clarity of major themes | Were major themes clearly presented in the findings? | Results section, pages 11-15 |
| 32 | Clarity of minor themes | Is there a description of diverse cases or discussion of minor themes? | Results section, pages 11-15 |

Adapted from Tong, A., et al. (2007). "Consolidated criteria for reporting qualitative research (COREQ): a 32-item checklist for interviews and focus groups." *Int J Qual Health Care* 19(6): 349-357.
